# “*I just want to be skinny*.”: A content analysis of tweets expressing eating disorder symptoms

**DOI:** 10.1371/journal.pone.0207506

**Published:** 2019-01-16

**Authors:** Patricia A. Cavazos-Rehg, Melissa J. Krauss, Shaina J. Costello, Nina Kaiser, Elizabeth S. Cahn, Ellen E. Fitzsimmons-Craft, Denise E. Wilfley

**Affiliations:** Department of Psychiatry, Washington University School of Medicine, St. Louis, Missouri, United States of America; Institut Català de Paleoecologia Humana i Evolució Social (IPHES), SPAIN

## Abstract

There is increasing concern about online communities that promote eating disorder (ED) behaviors through messages and/or images that encourage a “thin ideal” (i.e., promotion of thinness as attractive) and harmful weight loss/weight control practices. The purpose of this paper is to assess the content of body image and ED-related content on Twitter and provide a deeper understanding of EDs that may be used for future studies and online-based interventions. Tweets containing ED or body image-related keywords were collected from January 1-January 31, 2015 (N = 28,642). A random sample (n = 3000) was assessed for expressions of behaviors that align with subscales of the Eating Disorder Examination (EDE) 16.0. Demographic characteristics were inferred using a social media analytics company. The comprehensive research that we conducted indicated that 2,584 of the 3,000 tweets were ED-related; 65% expressed a preoccupation with body shape, 13% displayed issues related to food/eating/calories, and 4% expressed placing a high level of importance on body weight. Most tweets were sent by girls (90%) who were ≤19 years old (77%). Our findings stress a need to better understand if and how ED-related content on social media can be used for targeting prevention and intervention messages towards those who are in-need and could potentially benefit from these efforts.

## Introduction

There is emerging evidence that various online media platforms (e.g., blogs, social media) have content about ultra-thin beauty ideals that can be pervasive and easily accessible by even those who are relatively young in age (i.e., pre-adolescents). For instance, a 25-country European Kids Online survey found that 10% of children aged 9 to 16 had seen pro-ED sites online [1]. Related, one study examined 300 publically available images tagged as either thinspiration or thinspo (i.e., terms widely used when referring to thin-ideal content) on Twitter and Pinterest and found that their focus tended to be portrayals of “ultra-thin, bony, scantily-clad women” [2, 3]. A similar Twitter study identified 45 profiles via posts that contained the term “proana” ((i.e., a term that is short for pro-anorexia and often used to promote eating disorder [ED] behaviors) and found that consistent referencing of EDs (e.g., explicit mentions of bulimia and anorexia) occurred within these accounts and that most of these accounts acquired followers who themselves similarly posted about EDs [4]. A study examining posts on a pro-ED community on Reddit found that subscribers of this forum were consistently prompted by moderators to post pictures and updates about their disordered eating goals, and dialogue encouraging these behaviors tended to be the most frequent responses to these posts [5]. The burgeoning research is relevant for revealing the existence of online communities on social media that encourage and support disordered eating behaviors and/or candidly post about EDs.

To gain an even deeper understanding of the nature of online posts about disordered eating behaviors, researchers have examined how popular ED-associated hashtags and keywords may be distinguishable from one another by qualitatively coding and comparing their most popular themes. Results demonstrated that online posts which included the terms #thinspiration, #fitspo, and/or #fitspiration were qualitatively different from one another in how frequently they portrayed thin and objectified bodies and/or bone protrusions versus muscular, in-shape, and/or fit bodies [3, 6–9]. These studies are significant for dissecting pro-ED online content to a greater extent than prior studies and for helping to guide clinical interpretations of this type of content that has potentially concerning implications. An analysis of the clinical symptoms expressed in ED-related social media posts has not yet been accomplished but could likewise be beneficial to further understand the unique and systematic patterns of this type of postings. Such insights could increase understanding of how well this type of social networking aligns with the signs and symptoms that manifest in someone with an ED diagnosis and could additionally be used to inform clinical interpretations of this type of content.

In the present study our team examined ED-related content on Twitter and focused our content analysis on an examination of expressions consistent with ED symptoms as outlined by the Diagnostic and Statistical Manual of Mental Disorders (DSM) IV criteria for a clinical diagnosis to further the existing body of research [10]. Specifically, we used a validated measure that examines DSM-IV ED symptoms to guide our content analysis in order to examine how aligned social media posts are with clinical ED symptoms that would be expressed when describing symptoms of a diagnosable ED [11]. We hypothesize that tweets containing ED-related keywords will align with the shape, weight, and eating concerns that are defined with this scale. Studying this content on Twitter could have implications for future studies on ED identification, prevention, and treatment, specifically for young people who discuss facets of ED symptomatology online and may be more inclined to engage with similar, online-based interventions. This is especially important because coping with an ED is inherently difficult and stressful, and it can be further complicated because it can coincide with other conditions (e.g., depression and anxiety), making it difficult to determine the proper mode of treatment [12].

In addition to examining DSM ED criteria within tweets, we wanted to determine if a social analytics company could be used to successfully infer the demographics of Twitter users that would align with what the literature reports as individuals that have EDs. Consequentially, we used the services of DemographicsPro (http://www.demographicspro.com/) to infer the demographic characteristics of the individuals who tweeted about a shape, eating, or weight concern in our random sample of tweets. We expected DemographicsPro would identify that ED-related tweets are being posted by a young, female population that would mirror the highest risk group for EDs [4, 13, 14]. Identifying the characteristics of individuals who distribute pro-ED content on Twitter will allow for more targeted intervention strategies on social media.

## Methods

All the tweets used in the current study were publicly available. The Washington University Institutional Review Board classified our study as research that does not involve human subjects and thus it was not subject to institutional review board jurisdiction.

### Collection of tweets

In February 2015, body image or ED-related tweets in the English language were purchased from Gnip, Inc. (https://gnip.com/) for the full month of January (January 1-January 31, 2015) from the full Twitter data stream. To build a list of relevant keywords, we identified some ED-related words during a prior study of depression-related tweets [15] (e.g., thinspiration, thinspo, proana), and built upon this initial list by searching posts from ED/body image-focused Twitter accounts and searching for related terms on hashtagify.me (CyBranding, Ltd.), a search engine that finds the most popular synonymous hashtags (words preceded by # used to flag tweets about a specific subject) correlated with a searched term (hashtagify.me), and Topsy.com, a Twitter search engine that shows recent tweets with the keyword of interest. Topsy.com is no longer operational; however alternative sites such as socialert.net similarly allow users to search for tweets containing hashtags and/or keywords of interest. Terms that did not have a high specificity or were considered more apt to be used in ads were eliminated (e.g., skinny, thin, diet).

Our final list of body image or eating-disorder related search terms included 23 search terms: thinspo, #thinspo, #thinspos, thinspiration, #thinspiration, #bonespo, #edprobs, #EDproblems, #ednos, #edlogic, #proana, “pro ana”, #promia, “pro mia”, “hip bones”, #hipbones, “chest bones”, #chestbones, #collarbones, #bodyslip, #anamia, “ana/mia”, or “ana” and “mia”. The words “ana” and “mia” were only searched in combination (i.e., in the same tweet), but not when used alone due to their common use as first names. This list was provided to Gnip who then collected all tweets from January 2015 with these keywords of interest via the Historical PowerTrack API (http://support.gnip.com/apis/historical_api2.0/). See S1 Appendix for the filtering rules used for these keywords. After collecting tweets with these search terms, the research team scanned common retweets to identify and remove tweets that included our terms but were not explicitly related to our topic of interest (e.g., “Win a signed copy of Archer’s Voice from *Mia* Sheridan plus gift cards at *Ana*’s Attic”), resulting in a total of 28,642 tweets. From these ED-related tweets, 3,000 tweets were randomly selected for qualitative analysis using *proc surveyselect* in SAS version 9.4 (SAS Institute Inc. Cary, NC). A total of 3,000 tweets would allow estimation of the prevalence of tweets with specific themes with 95% confidence level with a margin of error of plus or minus < 2%. Data cannot be shared to comply with the Gnip\Twitter terms of service, but de-identified data for the codes assigned to the 3000 tweets, along with a codebook, are available within the Supporting Information files (S1 Dataset and S1 Codebook). Of the random sample of 3,000 tweets from the total sample of nearly 29,000 tweets, 2,584 (86%) were about EDs or body image. Approximately 416 (14%) were not discernibly about EDs or body image and were excluded from further analysis. Many of these excluded tweets used the term “hip bones” (e.g., “*I have big bruises on my hip bones and upper thighs”*, *“I have some hard ass hip bones”*).

### Themes

Because we had interest in discussions about body image/EDs on Twitter that could potentially be of clinical relevance (e.g., individuals expressing their own pro-ED behaviors/attitudes), we based the primary themes on the subscales from the Eating Disorder Examination (EDE) 16.0 [11]. The EDE is constructed from the DSM-IV ED diagnostic criteria and has been psychometrically validated as an assessment of ED psychopathology [16–18]. The three major themes based on the EDE 16.0 subscales included whether the tweet expressed a) shape concern (placing a high level of importance on or preoccupation with body image/shape), b) weight concern (placing a high level of importance on or preoccupation with body weight), and c) eating concern (having issues or preoccupation or guilt related to food/eating/calories, eating in secret, or fear of losing control over eating). Another subscale from the EDE 16.0, restraint, was included as a sub-code of the eating concern theme due to difficulty distinguishing between these two related codes in the context of tweets. Thus, when eating concern was an identified theme within a tweet, the tweet was further coded as restraint if the tweet specifically indicated avoiding or limiting food or calorie intake. Tweets with content relating to eating concern were also further coded as whether they specifically mentioned binging/purging of food.

**Fig 1:**
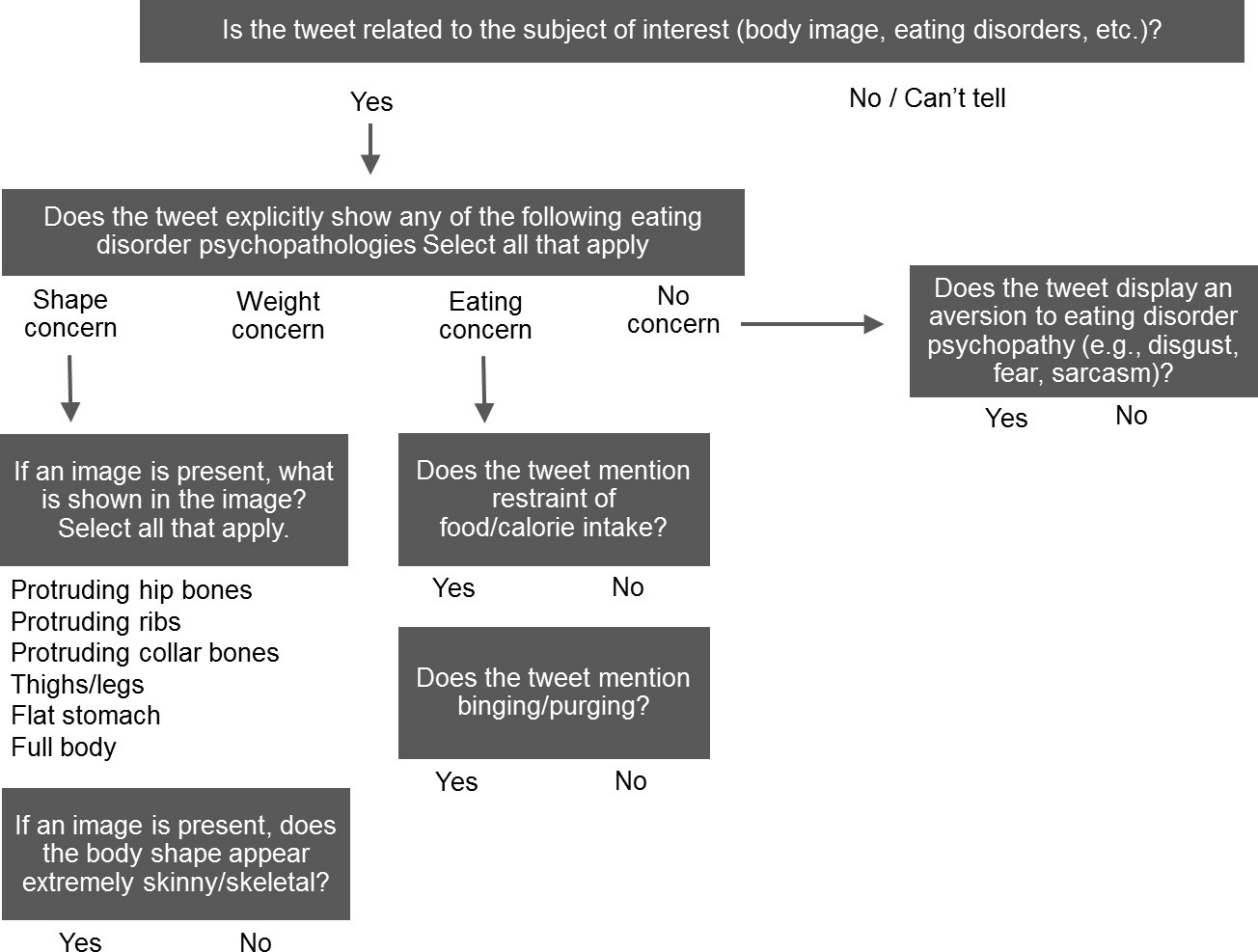
Flow of items to assess presence of eating disorder-related themes in tweets.

Based on the codes from a recent study of “thinspiration” tweets, tweets with content relating to a shape concern were further coded for the presence of an image and the type of body part displayed in the image [3]: protruding hip bones, protruding ribs, protruding collarbones, thighs/legs, flat stomach, full body. In addition, we also coded when the tweet went beyond showing a “thin ideal” to depict an extremely thin, starved, or skeletal body. Among tweets that did not display any of the subscales from the EDE 16.0, we coded whether tweets discouraged pro-ED content by expressing an aversion (i.e., disgust, fear, sarcasm) to such content on Twitter. Each tweet was defined as a codable unit. More than one theme could be coded for each tweet, except for tweets discouraging pro-ED content that were not coded for shape, weight, or eating concern. If a tweet contained a URL, the coder clicked on the link and coded content included on the landing page. Fig 1 shows the flow of the items used to assess the presence of the themes in each tweet.

### Coding of the tweets

Eight members of the research team were involved with coding for sufficient manpower to complete the coding in a timely manner. Coders were trained by discussing the codes described above with a senior member of the research team, reviewing example tweets for each theme, and then individually coding a practice set of 300 tweets. Codes for these tweets were compared among the coders to discuss discrepancies and develop a better understanding of the themes across all coders before coding the remaining tweets independently. Following the coding of the initial 300 tweets, each remaining tweet was coded independently by at least two coders. Inter-coder reliability for each theme across all coded tweets was assessed using Krippendorff’s α, which ranges from 0 to 1 with higher numbers indicating better coder agreement (α = 1 indicates perfect reliability) [19]. Most themes had high enough reliability to draw at least tentative conclusions (α≥0.667) [20]. Krippendorff’s α for the three major themes of interest based on the EDE 16.0 subscales were 0.75, 0.66, and 0.79 for shape concern, weight concern, and eating concern, respectively. Krippendorff’s α for displaying an extremely thin, starved, or skeletal body was 0.68. The median Krippendorff’s α for the remaining codes was 0.69 (range 0.60 to 0.88; only the codes for collarbones and thighs, depicting a full body, and restraint had α<0.67). However, all discrepancies were discussed between three main coders in order to resolve coding differences.

### Demographic characteristics of Twitter users

Twitter has been shown to be a valuable demographic inference tool. A 2012 analysis of Twitter users served as an initial step to enable inferences to be made about user demographics, concluding that more nuanced analyses on the biases in the Twitter population will enhance the ability for Twitter to be used as a sophisticated inference tool [21]. Using proprietary algorithms, DemographicsPro (http://www.demographicspro.com/) estimates or infers the likely demographic characteristics of Twitter users based on their Twitter use and behavior. The demographic predictions are based on multiple data signals, including the nature and strength of ties within Twitter networks, consumption of information on Twitter, and language used in tweets and biographies. Their methodologies have gone through iterative evaluation testing on established samples of Twitter users with verified demographics, with sample sizes ranging from 10,000 to 200,000 individuals depending on the demographic characteristic of interest to be inferred. A confidence of 95% or above is required by DemographicsPro to estimate a single demographic characteristic. Thus, out of 10,000 predictions, at least 9,500 would need to be accurate in order to accept the methodology used to make the prediction. For comparison purposes, DemographicsPro also provides demographic characteristics of typical Twitter users based on the Twitter median average, which is calculated from follower demographic characteristics across a sample of approximately 250,000 Twitter accounts from 10 million Twitter accounts analyzed.

## Results

### Tweet volume and keyword popularity

The most commonly used keyword was thinspo (13,450 tweets) followed by hipbones (4,829 tweets) and thinspiration (4,374 tweets) (of note, some tweets included more than one keyword). See Table 1 for frequencies of the ED/body image-related keywords. Approximately 43% of the collected tweets were original posts from the author while 57% were retweets. The median number of followers was 313 (inter-quartile range 111–876) and the total possible reach of these tweets (sum of all followers) was 35,310,650.

**Table 1:** Eating disorder or body image-related tweets by keyword, January 1 –January 31, 2015 (n = 28,642).

Keywords	**Number of Tweets**^**a**^
thinspo or #thinspo or #thinspos	13,450
“hip bones” or #hipbones	4,829
thinspiration or #thinspiration	4,374
#edprobs or #EDproblems	3,671
#proana or “pro ana”	1,424
anamia^b^	590
ednos	521
#edlogic	432
#bonespo	316
“chest bones” or #chestbones	281
#bodyslip	208
#collarbones	204
#promia or “pro mia”	100

^a^ Sum does not equal the total of tweets because some tweets include more than one term

^b^ Includes tweets with keywords “ana/mia”, “#anamia”, or “ana” and “mia” in any combination (i.e., in the same tweet)

### Tweets that displayed ED symptomology

Among the 2,584 ED related tweets, the most common symptom expressed was concern about body shape (65%, 1,679) (Table 2). Approximately 51% of these (861/1,679) included an image (the others either did not include an image or the link provided was a website that no longer could be viewed). A picture of the entire body was shown in 60% (514/861) of these images. The most commonly displayed specific body part was thin thighs/legs (273/861, 32%), followed by a flat stomach (248/861, 29%). Pictures displaying protruding bones were also commonly shown (hip bones 159/861, 18%; ribs 158/861, 18%; collar bones 123/861, 14%). Of the 861 shape concern tweets with an image, 51% (437/861) portrayed an extremely thin or skeletal person.

**Table 2:** Tweets expressing concern about body shape with an image (N = 861).

	n (%)
Image portrays an extremely thin or skeletal person	437 (51%)
Image focuses primarily on…	
Full body	514 (60%)
Thighs/legs	273 (32%)
Flat stomach	248 (29%)
Protruding hip bones	159 (18%)
Protruding ribs	158 (18%)
Protruding collar bones	123 (14%)

**Fig 2:**
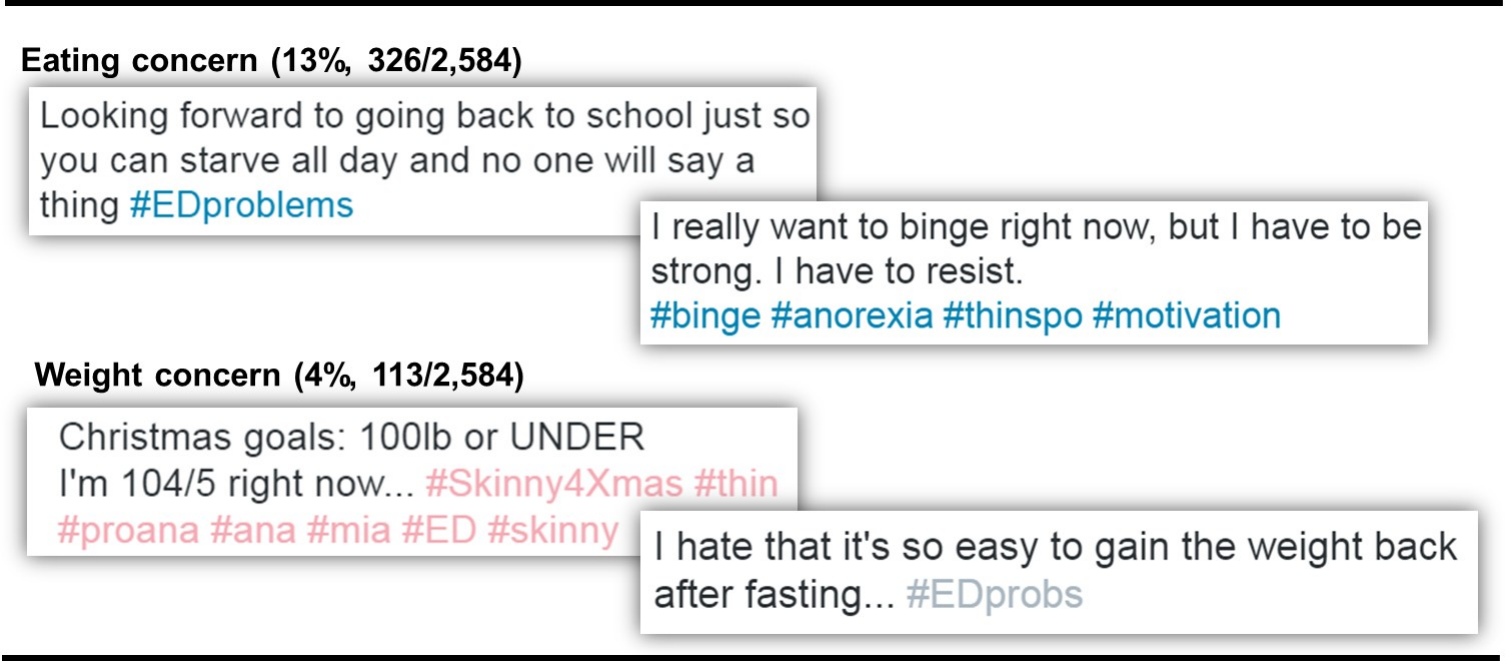
Tweets expressing concern about eating or weight.

Tweets expressing issues or preoccupation with food, eating, or calories (i.e., eating concern) were less common, constituting 13% (326) of the 2,584 ED-related tweets. Among these tweets, 54% (176/326) expressed engaging in restraint (limiting food/calories) and 28% (92/326) expressed engaging in binging and/or purging behavior. Weight concern (placing importance on actual body weight) was expressed in 4% (113/2,584) of the ED-related tweets. Examples of tweets expressing eating concern and weight concern are shown in Fig 2.

Only 23% (599) of the tweets did not express shape, eating, or weight concern or restraint, and among these, half (297, 50%) discouraged pro-ED tweets by expressing an aversion toward such content. Over half of these tweets (53%, 156) was a popular retweet that showed a selfie of an extremely thin young girl with the caption “*dk what's scarier*. *her protruding hip bones or the fact that this is people's idea of perfection*.” Notably, tweets that discouraged pro-ED tweets had a larger median number of retweets (median 12, range 0 to 2,845) than those that expressed shape, weight or eating concern (median 2, range 0 to 337) (Wilcoxon rank sum Z = 7.63, p<0.001). Similarly, individuals whose tweets discouraged pro-ED tweets had a larger median number of followers (median 309, range 0 to 270,059) than individuals who tweeted about shape, eating, or weight concerns (median 274, range 0 to 279,933) (Wilcoxon rank sum Z = 2.27, p = 0.024).

### Demographics of Twitter users

The 1,985 tweets expressing shape, eating, or weight concern were sent by 1,323 unique individuals. DemographicsPro, a social media analytics company, inferred the likely demographic characteristics of these individuals and provided demographic characteristics of typical Twitter users as a comparison. Results revealed that the 1,323 individuals who tweeted this content tended to be younger and a greater proportion female than the general Twitter audience. Specifically, 90% were female, compared to a Twitter median average of 54% female. Approximately 73% were age 17 to 19 years, 5% were ≤16 years old, 18% were age 20 to 24, and only 5% were age ≥25 compared to a Twitter median average of 31%, 1%, 34%, and 33% in these age ranges, respectively. The race/ethnicity of this sample was similar to that of the Twitter population, with 82% White, 16% African American, and 2% Hispanic, compared to Twitter median averages of 77%, 16%, and 7%, respectively.

## Discussion

We examined ED and body image-related tweets posted on Twitter for an in-depth study of their content, including text and images, focusing on investigating the extent to which tweets aligned with psychopathology assessed within the Eating Disorder Examination (EDE) 16.0 questionnaire [16–18]. While we cannot confirm that the tweets under study disclosed actual behavioral intent, our findings are nevertheless relevant for detecting tweets that are–for the most part–expressions of psychopathology corresponding with symptoms of an ED. Furthermore, our findings signaled that concerns about body shape which are a core feature of EDs [22–24] and can often challenge the recovery of EDs [22, 23] were the most common symptom being tweeted. Therefore, our findings are the first of its kind to reveal the detection of tweets that that are primarily expressions of ED indications.

Additionally, we found that the individuals sending the tweets were inferred to be girls in their teenage years, which is notably different than the average demographics of the general Twitter population. Our approach extends the work of Arseniev-Koehler et al. [4] who examined 45 pro-ED Twitter profiles by examining a broader sample of pro-ED content on Twitter for a month; in doing so, we validate their concerning finding that suggests that young girls are the bulk of the individuals distributing the pro-ED content on Twitter. The demographic traits of our tweeters correspond directly with related work that documents a tendency for individuals with EDs to be young women, with those aged 15–19 at particular risk for anorexia nervosa [25].

It is possible that some online communities provide individuals with EDs with a sense of belonging for those who feel isolated from their friends/family [26]. Although the extent to which our methods could be leveraged to target individuals who are struggling with ED-associated symptoms remains an open question, our findings signal promise for potentially leveraging social media as a tool for early identification of at-risk individuals who may benefit from ED-related prevention and intervention. There is a need for future research in this area especially in light of two recent studies that signal ED-related self-reported psychopathology and associated risks among women who were targeted for study recruitment via their socially networking online about pro-ED topics [27, 28].

Limitations should be considered when interpreting results from our study. Our list of keywords was not comprehensive of all body image or ED-related terms, especially prevention/treatment/clinical terms. Similarly, while *thigh gap* is a popular term on Twitter, it was not included in our study in order to hone in on ED and body-image related chatter rather than mainstream media or popular culture references (e.g., recent apology made by *Target* for photoshopping a model’s appearance to accentuate her thigh gap) [29, 30]. Tweets represent discussions on Twitter occurring during January, which is a time of the year when diet, exercise, and body image are often stressed. Additionally, we only included publicly available tweets in our sample, and it is possible that individuals tweeting from private accounts may generate different content. However, Twitter accounts default to a public setting [31], and the percentage of private Twitter accounts is very small [32]. The issue regarding privacy on social media is an emerging topic, but preliminary findings on Facebook (a traditionally more private social networking site), show that content posted does not differ based on account privacy settings [33]. Therefore, we have reason to believe that we would see similar results from tweets posted from private accounts. It is also impossible for us to determine the degree to which the tweets correlate with true ED psychopathology; future studies of this type are needed to assess the fidelity of our results. Some codes were more popular than others and it is possible that this is an artifact of the hashtags chosen. In addition, DemographicsPro infers demographic characteristics of Twitter users, and the possibility exists that the inferred characteristics of the Twitter users do not represent the actual Twitter users in our sample of tweets. Finally, some of the alpha coefficients representing reliability of code assignments were low and such codes can only be used to draw tentative conclusions.

Even with these limitations, we believe that this study supports the emerging literature on pro-ED content on social media by validating demographic information about the individuals posting this content and presenting new and intriguing findings regarding the alignment of this content with ED psychopathology. Our findings stress a need for continued research in this line of work, especially to better understand if and how this type of content can be used for targeting prevention and intervention messages towards those who are in-need and could potentially benefit from these efforts. Additionally, it would be beneficial for this study to be replicated on other social media platforms, specifically visually-dominant platforms such as Instagram, to see if the same ED psychopathology is observed. Most importantly, future research should aim to better understand how this type of content can be leveraged to find and reach individuals who are struggling with ED psychopathology.

## Supporting information

S1 Appendix Filtering rules for collecting ED-related tweets via Gnip. (DOCX)

S1 Dataset Codes assigned to the 3000 tweets used in the analysis. (CSV)

S1 Codebook Eating disorder/body image related tweets codebook. (DOCX)

## References

[1] Livingstone S., Haddon L., Görzig A., and Ólafsson K. *Risks and safety on the Internet*: *the perspective of European children* *Key findings from the EU Kids Online survey of 9–16 year olds and their parents in 25 countries*. LSE, London: EU Kids Online, 2010.

[2] CBS New York. Thinspiration: Doctors Concerned with Social Media Sites Promoting Eating Disorders CBS. 17 7 2012 Available from: http://newyork.cbslocal.com/2012/07/17/thinspiration-doctors-concerned-with-social-media-sites-promoting-eating-disorders/. Cited 4 Jan 2018.

[3] Ghaznavi J, Taylor LD. Bones, body parts, and sex appeal: an analysis of #thinspiration images on popular social media. Body Image. 2015;14:54–61. 10.1016/j.bodyim.2015.03.006 25880783

[4] Arseniev-Koehler A, Lee H, McCormick T, Moreno MA. #Proana: pro-eating disorder socialization on Twitter. J Adolesc Health. 2016;58(6):659–64. 10.1016/j.jadohealth.2016.02.012 27080731

[5] Sowles SJ, McLeary M, Optican A, Cahn E, Krauss MJ, Fitzsimmons-Craft EE, Wilfley DE, Cavazos-Rehg PA. A content analysis of an online pro-eating disorder community on Reddit. Body Image. 2018;24:137–144. 10.1016/j.bodyim.2018.01.001 29414146 PMC5869127

[6] Tiggemann M, Churches O, Mitchell L, Brown Z. Tweeting weight loss: a comparison of #thinspiration and #fitspiration communities on Twitter. Body Image. 2018;25:133–138. 10.1016/j.bodyim.2018.03.002 29567619

[7] Talbot CV, Gavin J, van Steen T, Morey Y. A content analysis of thinspiration, fitspiration, and bonespiration imagery on social media. J Eat Disord. 2017;5(1):40 10.1186/s40337-017-0170-2 29021900 PMC5613385

[8] Tiggemann M, Zaccardo M. ‘Strong is the new skinny’: a content analysis of #fitspiration images on Instagram. J Health Psychol. 2016:OnlineFirst. 10.1177/1359105316639436 27611630

[9] Carrotte ER, Prichard I, Lim MSC. “Fitspiration” on social media: a content analysis of gendered images. J Med Internet Res. 2017;19(3):e95 10.2196/jmir.6368 28356239 PMC5390113

[10] American Psychiatric Association. Diagnostic and statistical manual of mental disorders, 4th ed Washingtion, DC: American Psychiatric Association Publishing; 2000.

[11] Fairburn CG, Beglin SJ. Assessment of eating disorders: interview or self-report questionnaire? Int J Eat Disord. 1994;16(4):363–70. 7866415

[12] Mishra R. The case: starving for perfection. Camb Q Healthc Ethics. 2012;21(3):396–7. 10.1017/S0963180112000138 22624544

[13] Qian J, Hu Q, Wan Y, Li T, Wu M, Ren Z, et al Prevalence of eating disorders in the general population: a systematic review. Shanghai Arch Psychiatry. 2013;25(4):212–23. 10.3969/j.issn.1002-0829.2013.04.003 24991159 PMC4054558

[14] Swanson SA, Crow SJ, Le Grange D, Swendsen J, Merikangas KR. Prevalence and correlates of eating disorders in adolescents: results from the National Comorbidity Survey Replication Adolescent Supplement. Arch Gen Psychiatry. 2011;68(7):714–23. 10.1001/archgenpsychiatry.2011.22 21383252 PMC5546800

[15] Cavazos-Rehg PA, Krauss MJ, Sowles S, Connolly S, Rosas C, Bharadwaj M, et al A content analysis of depression-related tweets. Comput Human Behav. 2016;54:351–7. 10.1016/j.chb.2015.08.023 26392678 PMC4574287

[16] Berg KC, Peterson CB, Frazier P, Crow SJ. Psychometric evaluation of the eating disorder examination and eating disorder examination-questionnaire: a systematic review of the literature. Int J Eat Disord. 2012;45(3):428–38. 10.1002/eat.20931 21744375 PMC3668855

[17] Mond JM, Hay PJ, Rodgers B, Owen C, Beumont PJV. Validity of the eating disorder examination questionnaire (EDE-Q) in screening for eating disorders in community samples. Behav Res Ther. 2004;42(5):551–67. 10.1016/S0005-7967(03)00161-X 15033501

[18] Peterson CB, Crosby RD, Wonderlich SA, Joiner T, Crow SJ, Mitchell JE, et al Psychometric properties of the eating disorder examination-questionnaire: factor structure and internal consistency. Int J Eat Disord. 2007;40(4):386–9. 10.1002/eat.20373 17304585

[19] Krippendorff K. Computing Krippendorff's alpha reliability; 2007. Available from: https://repository.upenn.edu/asc_papers/43/. Cited 4 Jan 2018.

[20] Krippendorff K. Reliability in content analysis. Hum Commun Res. 2004;30(3):411–33. 10.1111/j.1468-2958.2004.tb00738.x

[21] Mislove A, Lehmann S, Ahn Y-Y, Onnela J-P, Rosenquist JN. Understanding the demographics of Twitter users. Proceedings of the Fifth International AAAI Conference on Weblogs and Social Media; 2011 July. Barcelona, Spain. AAAI Press; 2011. p. 554–7. Available from: https://www.aaai.org/ocs/index.php/ICWSM/ICWSM11/paper/view/2816/3234.

[22] Cooper PJ, Fairburn CG. Confusion over the core psychopathology of bulimia nervosa. Int J Eat Disord. 1993;13(4):385–9. 8490640 10.1002/1098-108x(199305)13:4<385::aid-eat2260130406>3.0.co;2-w

[23] Fairburn CG. Cognitive behavior therapy and eating disorders. New York: The Guilford Press; 2008.

[24] Fairburn CG, Garner DM. The diagnosis of bulimia nervosa. Int J Eat Disord. 1986;5(3):403–19.

[25] Smink FR, van Hoeken D, Hoek HW. Epidemiology of eating disorders: incidence, prevalence and mortality rates. Curr Psychiatry Rep. 2012;14(4):406–14. 10.1007/s11920-012-0282-y 22644309 PMC3409365

[26] Pater JA, Haimson OL, Andalibi N, Mynatt ED. “Hunger hurts but starving works”: characterizing the presentation of eating disorders online. Proceedings of the 19th ACM Conference on Computer-Supported Cooperative Work & Social Computing; 2016 Feb; San Francisco, California, USA. New York, ACM Publications; 2016. p. 1185–200. Availabe from: https://dl.acm.org/citation.cfm?id=2820030.

[27] Holland G, Tiggemann M. “Strong beats skinny every time”: disordered eating and compulsive exercise in women who post fitspiration on Instagram. Int J Eat Disord. 2017;50(1):76–79. 10.1002/eat.22559 27302867

[28] Cornelius T, Blanton H. The limits to pride: A test of the pro-anorexia hypothesis. Eating disorders. 2016;24(2):138–147. 10.1080/10640266.2014.1000102 25658245

[29] Brown A. Picture [im]perfect: photoshop redefining beauty in cosmetic advertisements, giving false advertising a run for the money. Tex Rev Ent & Sports L. 2015;16(2):87–106.

[30] M. C. How the media is brainwashing our perception of beauty. 2015 March 8 [cited 4 Jan 2018]. Available from: http://www.teenink.com/opinion/social_issues_civics/article/778057/How-the-Media-is-Brainwashing-Our-Perception-of-Beauty/.

[31] Twitter. About public and protected tweets. 2018 [cited 3 Januray 2018]. Available from: https://help.twitter.com/en/safety-and-security/public-and-protected-tweets.

[32] Beevolve. An exhaustive study of Twitter users across the world. 2012 Oct 10 [cited 3 January 2018]. Available from: http://www.beevolve.com/twitter-statistics/.

[33] Fiesler C, Dye M, Feuston JL, Hiruncharoenvate C, Hutto CJ, Morrison S, et al. What (or who) is public?: privacy settings and social media content sharing. Proceedings of the 2017 ACM Conference on Computer Supported Cooperative Work and Social Computing; 2017 Feb; Portland, Oregon, USA. New York, ACM Publications; 2017. p. 567–80. Availabe from: https://dl.acm.org/citation.cfm?id=2998223.

