# Supplementary material for: “I just want to be skinny.”: A content analysis of tweets expressing eating disorder symptoms
Source: PLoS One. 2019 Jan 16;14(1):e0207506. doi: 10.1371/journal.pone.0207506 (PMC6334988; doi:10.1371/journal.pone.0207506)
Supplement: S1 Appendix — (DOCX) [file pone.0207506.s001.docx]

**S1 Appendix.** Filtering rules for collecting ED-related tweets via Gnip

(#thinspo OR #thinspiration OR thinspo OR “hip bones” OR #EDproblems OR #proana OR #edprobs OR thinspiration OR #hipbones OR “chest bones” OR #ednos OR #collarbones OR #edlogic OR #bonespo OR #promia OR #bodyslip OR #thinspos OR “ana/mia” OR #anamia OR #chestbones) lang:en

(“pro mia” –Clarisonic) lang:en

(ana mia) lang:en

(“pro ana” –breakfast) lang:en
