# Supplementary material for: “I just want to be skinny.”: A content analysis of tweets expressing eating disorder symptoms
Source: PLoS One. 2019 Jan 16;14(1):e0207506. doi: 10.1371/journal.pone.0207506 (PMC6334988; doi:10.1371/journal.pone.0207506)
Supplement: S1 Codebook — (DOCX) [file pone.0207506.s003.docx]

Eating Disorder(ED) / Body Image Related Tweets

**Project contact person:** Melissa Krauss, mkrauss@wustl.edu

**Project description:** Random sample of body image or eating disorder(ED)-related tweets.

**Sample procedures:** Body image or ED-related tweets in the English language were purchased from Gnip, Inc. for January 1-31, 2015 from the full Twitter data stream. These tweets contained specific body image or ED-related keywords which can be found in the methods section of the accompanying article. 3000 of these tweets were randomly sampled for qualitative analysis.

**Sample size:** n=3000

**Data collection time period:** January 1-31, 2015

| **Variable name** | **Variable label** | **Universe** | **Code** | **N** |
| --- | --- | --- | --- | --- |
| id | Unique identifier (not actual TweetID) | All tweets | NA | 3000 |
| ed_about | Was the tweet related to ED/body image? | All tweets | 0=no  1=yes  9=unk | 342  2584  74 |
| shape | Tweet expressed shape concern | Tweets related to ED/body image (ed_about=1) | 0=no  1=yes  =not in universe | 905  1679  416 |
| noimage | No image in tweet | Shape concern tweets (shape=1) | 0=image  1=no image  =not in universe | 861  818  1321 |
| body | Image showed entire body | Shape concern tweets (shape=1) with an image (noimage=0) | 0=no  1=yes  =not in universe | 347  514  2139 |
| thighs | Image showed thighs | Shape concern tweets (shape=1) with an image (noimage=0) | 0=no  1=yes  = not in universe | 588  273  2139 |
| stomach | Image showed stomach | Shape concern tweets (shape=1) with an image (noimage=0) | 0=no  1=yes  = not in universe | 613  248  2139 |
| hipbones | Image showed hip bones | Shape concern tweets (shape=1) with an image (noimage=0) | 0=no  1=yes  = not in universe | 702  159  2139 |
| ribs | Image showed ribs | Shape concern tweets (shape=1) with an image (noimage=0) | 0=no  1=yes  = not in universe | 703  158  2139 |
| collarbones | Image showed collarbones | Shape concern tweets (shape=1) with an image (noimage=0) | 0=no  1=yes  = not in universe | 738  123  2139 |
| extreme | Image showed extremely thin or skeletal person | Shape concern tweets (shape=1) with an image (noimage=0) | 0=no  1=yes  = not in universe | 424  437  2139 |
| eating | Tweet expressed eating concern | Tweets related to ED/body image (ed_about=1) | 0=no  1=yes  = not in universe | 2258  326  416 |
| restraint | Tweet expressed restraint (limiting food/calories) | Eating concern tweets (eating=1) | 0=no  1=yes  = not in universe | 150  176  2674 |
| purge | Tweet expressed engaging in binging or purging behavior | Eating concern tweets (eating=1) | 0=no  1=yes  = not in universe | 234  92  2674 |
| weight | Tweet expressed weight concern | Tweets related to ED/body image (ed_about=1) | 0=no  1=yes  =not in universe | 2471  113  416 |
| noconcern | Tweet did not express eating, weight, or shape concerns | Tweets related to ED/body image (ed_about=1)’ | 0=concern expressed  1=no concern expressed  = not in universe | 1985  599  416 |
| aversion | Tweet expressed aversion to pro-ED content | Tweets that did not express eating, weight or shape concerns (noconcern=1) | 0=no  1=yes  = not in universe | 302  297  2401 |
